# Effective Nanoparticle-Based Nasal Vaccine Against Latent and Congenital Toxoplasmosis in Sheep

**DOI:** 10.3389/fimmu.2020.02183

**Published:** 2020-09-09

**Authors:** Céline Ducournau, Nathalie Moiré, Rodolphe Carpentier, Pauline Cantin, Clément Herkt, Isabelle Lantier, Didier Betbeder, Isabelle Dimier-Poisson

**Affiliations:** ^1^Team BioMAP, Université de Tours, INRAE, Tours, France; ^2^INFINITE, Institute for Translational Research in Inflammation, University of Lille, Inserm, Lille, France; ^3^Vaxinano, SAS, Lille, France; ^4^INRAE, Université de Tours, Tours, France

**Keywords:** ovine toxoplasmosis, nanoparticle, nasal vaccination, adjuvant free, one-health approach

## Abstract

*Toxoplasma gondii* is a parasitic protozoan of worldwide distribution, able to infect all warm-blooded animals, but particularly sheep. Primary infection in pregnant sheep leads to millions of abortions and significant economic losses for the livestock industry. Moreover, infected animals constitute the main parasitic reservoir for humans. Therefore, the development of a One-health vaccine seems the best prevention strategy. Following earlier work, a vaccine constituted of total extract of *Toxoplasma gondii* proteins (TE) associated with maltodextrin nanoparticles (DGNP) was developed in rodents. In this study we evaluated the ability of this vaccine candidate to protect against latent and congenital toxoplasmosis in sheep. After two immunizations by either intranasal or intradermal route, DGNP/TE vaccine generated specific Th1-cellular immune response, mediated by APC-secretion of IFN-γ and IL-12. Secretion of IL-10 appeared to regulate this Th1 response for intradermally vaccinated sheep but was absent in intranasally-vaccinated animals. Finally, protection against latent toxoplasmosis and transplacental transmission were explored. Intranasal vaccination led to a marked decrease of brain cysts compared with the non-vaccinated group. This DGNP/TE vaccine administered intranasally conferred a high level of protection against latent toxoplasmosis and its transplacental transmission in sheep, highlighting the potential for development of such a vaccine for studies in other species.

## Introduction

*Toxoplasma gondii*, an obligate intracellular parasitic protozoan, is the causative agent of toxoplasmosis. This parasitic zoonosis is a widespread, infectious disease of warm-blooded vertebrates (particularly sheep), in which congenital infection may result in abortion or stillbirth. Sheep infection occurs after ingestion of oocysts shed by cats in the environment (1, 2). Seroprevalence in sheep depends on the country and can reach up to 98% (Egypt) (3), while European studies report 74% and 87% seroprevalence in the United Kingdom (UK) and Belgium, respectively (4, 5). In France, the global seroprevalence in adult sheep is estimated to be about 89%, and 81% in carcasses for consumption (6). According to the Advisory Committee on the Microbiological Safety of Food, in a report published by the Food Standards Agency in 2012, human toxoplasmosis is the second major cause of death due to foodborne illness in United States – 24% *vs* 28% for *Salmonella spp.* (7) – and 30% of human infections are due to sheep meat consumption (8). More importantly, human worldwide seroprevalence of toxoplasmosis is estimated to be about 37%, ranging from 4% in South Korea (9) to 84% in Madagascar (10).

Toxoplasmosis is a multifaceted disease. Initial contact with the parasite triggers a protective immune response in immunocompetent animals or humans with no, or only few, symptoms. In immunodeficient humans, clinical signs define the acute phase of infection. By contrast, chronic infection is characterized by the persistence of the parasite as dormant cysts. These cysts can be located in the muscles, brain or eyes, leading to neuropathies, or ocular toxoplasmosis (2). In sheep, acute toxoplasmosis is characterized by intermediate symptoms, unlike other either more (marsupials, new world monkeys) or less sensitive (pigs, cattle) species (11–14). However, primary infection during gestation leads to serious congenital damage, the severity depending on the stage of gestation. Most of the time, infection at an early gestational stage causes abortion, while delivery of sub-clinical newborns is typical of infection in later stages (15, 16). In addition to abortions due to *T. gondii*, pregnant sheep were affected by physiological features characterizing an acute phase infection. Two main hypotheses are proposed: the first one is that infection is followed by hyperthermia leading to abortion because of fetal hypoxia, up to 1 month before delivery (17, 18); the second one, is due to placental thrombosis and vascular damage, independent of hyperthermia, or parasitic replication (19). In the United Kingdom, that breeds 30% of European sheep, toxoplasmosis is responsible for over 0.5 M lamb deaths each year, resulting in an estimated $16 – 32 M losses for the livestock industry (8).

To fight ovine toxoplasmosis there exists only one commercial vaccine: a live, intramuscular delivery vaccine from the S48 strain, Ovilis Toxovax^®^ (MSD Animal Health) (20), which is available in only some countries and only partially protects ewes during gestation since about 76% of offspring from vaccinated sheep are viable *vs* 18% from non-vaccinated ewes (21). However, this vaccine causes adverse effects, has a short shelf-life and is not suitable for human use (22). For many years, strategies using sub-unit vaccine in sheep failed to protection against infection. Already in 1989, Buxton et al. vaccinated sheep sub-cutaneously with surface antigens of *T. gondii* incorporated into ISCOMs (an Immuno-Stimulating Complex of antigens and adjuvants), but without success (23). More recently, a total extract of tachyzoites was encapsulated into PLG [a poly(lactide-co-glycolide) polymer] micro- and nano-particles adjuvanted with cholera toxin and used to immunize sheep *via* the intranasal route. Despite induction of humoral and cellular immune responses, no protection was observed (24). Other methods were explored, especially DNA vaccination that showed specific humoral responses and cytokine secretions but without conferring any protection (25–27). Nonetheless, vaccination remains the best option for protection considering the strong cellular immune response induced upon infection. Following ovine infection, IFN-γ-producing CD4+ and CD8 + T cells are detected in efferent lymph and the protective Th1 response becomes established in the long term (28, 29). More recently, early T-cell-independent production of IFN-γ and IL-12 has been highlighted and permitted the switch to an antigen-specific response around 8 days after infection (30). These immune mechanisms strongly contribute to protection against subsequent infection, especially during gestation (31). Ovine toxoplasmosis is therefore a major problem in veterinary as well as public health and, because of the only partial effectiveness of current vaccines, the development of a new and efficient one-health strategy of vaccination is urgently needed. The one health approach is a transdisciplinary approach taking into account the infection and its consequence on the animal and human health and also, on the environment at the local and global levels. The one health response to control toxoplasmosis using vaccination will aim to prevent congenital disease in both humans and target animals, to produce safe meat for human consumption and to reduce environmental contamination with oocysts (32, 33).

Over several years, we have been developing a nanoparticle-based vaccine able to deliver antigens of *T. gondii* (TE) and to be administered by the mucosal route in order to reproduce, as closely as possible, the natural infection route. The nanoparticle (DGNP) is composed of maltodextrin with a phospholipid core and is able to carry large amounts of antigens (34). DGNP interactions with airway mucosa and antigen delivery to mucosal cells have been previously described and showed that DGNP increased the nasal residence time and protein delivery into cells (35–37). In addition to display a long-term stability, this vaccine has been shown to induce a robust immune response after intranasal vaccination against acute (100% survival), latent (70% reduction in cerebral load) and congenital (86% reduction in fetal load) toxoplasmosis in mice, associated with a *Toxoplasma* specific Th1/Th17 cellular immune response (38, 39). This One-Health approach to a safe and promising vaccine led us to assess its efficiency in ovine toxoplasmosis. The strikingly-improved protection conferred by our vaccine using the nasal route of administration is related to a huge reduction of parasite loading in the chronic phase or during the transplacental transmission to the fetus.

## Materials and Methods

### Animals and Ethics Statements

Eight-to-ten month-old ewes for experimental procedures were selected from an INRAE Nouzilly livestock flock for their negative abortive-pathogen status: *T. gondii*, *Neospora caninum*, *Brucella sp.*, *Coxiella burnetii*, and *Chlamydophila abortus*. The Préalpes du Sud breed was chosen for its low prolificity (1.43). Sheep were housed at the Infectiology Plateform (PFIE) INRAE, Nouzilly, in accordance with the guidelines for animal experimentation (EU Directive 2010/63/EU), and the protocol was approved by both the local ethics committee (CEEA VdL), and the Ministry of Education and Research.

### DGNP Synthesis Labeling and *ex vivo* Tracking

Cationic and porous maltodextrin-based nanoparticles with a lipid core (DGNP) were produced, labeled with DiR (1,1′-Dioctadecyl-3,3,3′,3′-Tetramethylindotricarbocyanine Iodide, ThermoFisher) and characterized, as previously described (36).

Sheep were inoculated with these DGNP-DiR (600 μg) as described below (*section administration of the Vaccine*) by the intranasal or the intradermal route (in the neck or in the cheek). 1 and 24 h after inoculation, one sheep per group was sacrificed to sample inoculation sites, spleen and relevant efferent lymph nodes (submaxillary, parotid, retropharyngeal, palatine, and pharyngeal tonsils, prescapular, mediastinal, and mesenteric). The DGNP-DiR fluorescence in *ex vivo* samples was observed using the pre-clinical *in vivo* imaging system IVIS^®^ Spectrum (PerkinElmer). Tissue samples were imaged under epi-illumination with the filters set at 745 nm excitation/800 nm emission wavelengths. The DiR intensity was expressed as an average radiant efficiency.

### Preparation and Administration of the Vaccine

*Toxoplasma gondii* tachyzoites from the type I RH strain, grown in human foreskin fibroblasts, were killed by freezing. Total extract of antigens (TE) were obtained by freeze-thaw cycles and sonications as previously described (38). Protein concentration was determined using microBCA reagent (Pierce). The DGNP nanoparticles, made of a reticulated maltodextrin matrix with a lipid core, were prepared as previously described (38). The vaccine was prepared by mixing 600 μg of DGNP with 200 μg of antigens (TE). Intranasal administration was performed with 200 μL using an automatic syringe (Vital Concept Agriculture, FRANCE) with an atomizer LMA MADgic (Telefex, Ireland) to spray micro-droplets behind the nasal turbinates in order to target nasal associated lymphoid tissues. For intradermal injection, 1 mL was administered using an Agro-Jet MIT II needle-free injector (Medical International Technologies Inc. Canada) with a six-point head to inoculate the vaccine through the skin. Micro-thin jets dispersed the inoculum into the soft tissue thanks to 10-bar of pressure on the neck or the cheek.

### Experimental Design

**FIGURE 1:**
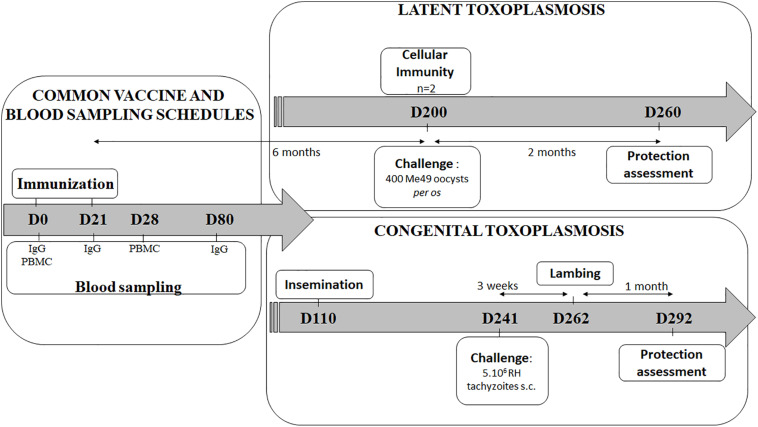
Vaccination and challenge overview of the latent and the congenital experiments. Experiment day are indicated as numbers (D0-D292). The vaccine schedule is common for both experiments (left): two immunizations 21 days apart (D0, D21), blood sampling for IgG detection by ELISA (D0, D21, and D80), and for PBMC (peripheral blood mononuclear cells) collection for antigen recall assay (D0, D28). In latent experiment (upper right): 6 months after vaccination (D200), spleen and lymph nodes from 2 sheep were used for cellular immune response study, and remaining animals were *per os* challenged then sacrifice 2 months for brain parasitic load counting. In congenital experiment (lower right): sheep were artificially inseminated 3 months after immunizations (D110), then s.c. challenge (D241) was performed 3 weeks before lambing while protection was evaluated 1 month after lambing by determining the sheep and lamb brain cyst load (D292).

In order to evaluate the vaccine’s effectiveness against latent toxoplasmosis and against *Toxoplasma* transplacental transmission, two independent experiments were conducted (Figure 1).

#### Protection Against Latent Toxoplasmosis

Control animals were administered with blank DGNP (group CTL, *n* = 12, half by intranasal route and half by intradermal route). Three groups were immunized by either the intranasal route (group IN, *n* = 12), the intradermal route in the neck area (group IDn, *n* = 12) or the intradermal route in the cheek area (group IDc, *n* = 12). All ewes were inoculated twice, 3 weeks apart (D0 and D21), and then infected orally with 400 sporulated oocysts of the type II Me49 strain (donated by Aurélien Dumètre, IHU-Méditerranée Infection) 6 months after the prime. Ten ewes were sacrificed 2 months after the challenge to study the conferred protection, except two animals per group were sacrificed before challenge to analyze the cellular response.

#### Protection Against Transplacental Transmission

Control animals were administered with blank DGNP (group CTL, *n* = 12, half by intranasal route and half by intradermal route). Two groups were immunized by either the intranasal route (group IN, *n* = 12) or the intradermal route in the neck area (group IDn, *n* = 12). A last group was experimentally primary infected as protected control (group INF, *n* = 12). Groups CTL, IN and IDn were inoculated twice, 3 weeks apart (D0 and D21) while group INF ewes were experimentally infected only once at D21 with 100 sporulated oocysts of type II Me49 strain *per os*. 3 months after vaccination, artificial insemination was carried out in the natural mating period and after hormonal synchronization. All ewes in all groups were subcutaneously challenged with 5.10^6^ tachyzoites of type I RH strain 3-weeks-prior to lambing. Ewes and lambs were sacrificed 1 month after lambing to study the transplacental passage of tachyzoites.

### *In vitro* Immunostimulatory Properties of the Vaccine

In order to evaluate the capacity of the vaccine formulation to stimulate splenocytes *in vitro*, spleens were collected from 4 sheep obtained at the slaughterhouse (INRAE Nouzilly, France). Spleens were dissociated through a nylon mesh to remove tissue debris and to generate a single-cell suspension. Hypotonic shock (sterile water) was used to remove erythrocytes. According to Steinman et al., with some modifications (40), adhesion steps from spleen single-cell suspension were performed to obtain lymphocyte-enriched population (non-adherent cells) and APC-enriched cells (adherent cells). The cells were characterized by flow cytometry before *in vitro* restimulation: 1.10^6^ cells were seeded in 24-well plates in 1 ml of culture medium and then incubated with 5 μg/ml TE, 15 μg/ml DGNP, or the DGNP/TE vaccine. Supernatants were harvested and assayed for IFN-γ, IL-12, IL-17, and IL-10 after a 72 h-culture period.

### Humoral Immune Response After Vaccination

As described previously (41), *Toxoplasma*-specific IgG were investigated by endpoint ELISA at day 0, 3 weeks after the first vaccination (D21), 2 months after the second vaccination (D80), and finally, 2 months after infection. Briefly, wells were coated with 10 μg/ml TE and IgG presence in serum samples (serial 2-fold dilution from 1/25 to 1/51200) was revealed with a donkey anti-sheep IgG, alkaline-phosphatase-conjugated and diluted to 1:5000 (Jackson Immunoresearch), followed by addition of 100 μl/well of a 1 mg/ml pNPP solution (Sigma Aldrich). Optical density was measured at 405 nm on a microtiter plate reader (Biotek Instruments). The positive threshold was calculated independently for each assay using the mean of the optical density of the same negative sera to which was added 2.5 standard deviations and related to blank.

### Cellular Immune Response After Vaccination

Peripheral Blood Mononuclear Cells (PBMC) of all ewes, from both experiments, were isolated at day 0 and day 28 (7 days after the last vaccination) using Ficoll Histopaque^®^-1077 (Sigma Aldrich). Spleen and peripheral lymph nodes (submaxillary, parotid, retropharyngeal, pharyngeal and palatine tonsils, prescapular, mediastinal, and mesenteric) were collected 6 months after the first immunization from two ewes per group of the latent experiment. PBMC and single-cell-suspensions from lymph nodes were prepared by seeding, in triplicate, 3.10^5^ cells in 96-well culture plates that were stimulated with 20 μg/ml TE, or 10 μg/ml Concanavalin A as a positive control. Cellular proliferation was assayed with colorimetric-proliferation ELISA (BrdU, Roche) after 48 h; supernatants were harvested and assayed for IFN-γ, IL-12, IL-17, and IL-10 after 72 h, as described below.

### Flow Cytometry Staining

Spleen and lymph node cells (5.10^5^) were seeded in 96-well plates in 200 μL PBS containing 5% Fetal Calf Serum (PBS-FCS) for 15 min at 4°C, and then stained in the same medium. After centrifugation, antibodies were added for 30 min at 4°C in a final volume of 100 μl at the recommended dilutions. After two washes in PBS-FCS, cells were resuspended in 200 μl PBS for analysis. For each sample, at least 10,000 events were acquired and analyzed using the Flowlogic software (Miltenyi Biotec).

Purified antibodies and hybridoma supernatants were used to characterize the splenocytes, lymphocytes and APC populations. Anti-sheep CD4 (clone 44.38), anti-sheep CD8 (clone 38.65), anti-sheep MHC class II DQ DR polymorphic (clone 28.1), anti-human CD14 (clone Tük4), anti-bovine CD11b (clone CC126; all from Serotec), and anti-bovine CD11c (BAQ153A; Kingfisher Biotech, inc.) were used following the manufacturer’s recommendations. The anti-ruminant DU204 (kindly provided by I. Schwartz-Cornil) was used, diluted twice. Matched isotype controls were used at the appropriate dilutions.

### Cytokine Assays

IFN-γ, IL-12, IL-17, and IL-10 assays were performed by ELISA. Briefly, wells were coated with 2 μg/ml of capture antibodies (mouse anti-bovine IFN-γ, IL-12, or IL-10; AbD Serotec/Bio-Rad Antibodies or rabbit, anti-bovine IL-17; KingFisher Biotech, Inc.). Recombinant bovine IFN-γ (Perbio), bovine IL-12 (KingFisher Biotech, Inc.), ovine IL-17 (KingFisher Biotech, Inc.), bovine IL-10 (AbD Serotec/Bio-Rad Antibodies) were used as standards and cytokines were revealed by addition of 100 μl/well of detection antibodies at 1 μg/ml (biotin conjugated mouse anti-bovine IFN-γ, IL-12, or IL-10; AbD Serotec/Bio-Rad Antibodies) or 0.1 μg/ml biotin conjugated rabbit anti-bovine IL-17 (KingFisher Biotech, Inc.), followed by addition of Extravidin/HRP and TMB substrate (Sigma Aldrich). Reactions were stopped by addition of 1 M sulfuric acid and optical density was measured at 450 nm on a microtiter plate reader (Biotek Instruments).

### Detection of *Toxoplasma* in Brain

To evaluate the protective efficacy of the vaccine, the parasitic load was determined in brain samples using two methods: direct cyst count and the presence of *Toxoplasma* DNA by nested PCR.

#### Cyst Count

Brains were homogenized in PBS containing 100 U/ml penicillin and 100 μg/ml streptomycin, as previously described (41). After washing, samples were layered on 5 ml of 0.9% NaCl/90% Percoll (Sigma) and interfaces containing cysts were recovered, mixed with corresponding pellets from upper phases, washed, and resuspended in PBS. Cysts were counted using a microscope and the detection limit was determined for each animal related to its own sample volume.

#### DNA Extraction and PCR

Brain samples (125 mg/ml buffer) were mixed in lysis buffer (Tris 50 mM pH 8, EDTA 50 mM, 1% SDS, and 50 μg/ml of proteinase K) using Gentlemacs and incubated overnight at 56°C. DNA from 25 mg of tissue in 200 μL of lysis buffer was then extracted using the NucleoSpin Tissue kit following the manufacturer’s instructions (Macherey-Nagel SARL, France).

Nested PCR targeting the 18S region was used to detect *T. gondii* DNA. Briefly, each 25 μl reaction contained 12.5 μl Go Taq green master mix (Promega) and 250 ng DNA. First-round reactions contained 0.2 μM of forward (5′-AACGT TCATGCTTGACTTCTC-3′) and reverse (5′-CAAGGTTAT AAACTCGTTGG-3′) primers, reactions were made to a final volume of 25 μl with DNase/RNase free water. A positive (cDNA of *T.* gondii) and a negative control (dH2O) were included. Cycling conditions for the two rounds of amplification were 2 min at 95°C, followed by 40 cycles of 45 s at 95°C, 30 s at 52°C, 1 min at 72°C, and a final extension period of 5 min at 72°C. Five microliters of the first round PCR products were used in the second-round reaction with the following primers: internal forward (5′-CACCAGGTCCAGACATAGGA-3′) and reverse (5′-AAGAACACGAAGTTCCTGATC-3′) primers. The nested PCR products were electrophoresed on a 2% (w/v) agarose gel and visualized under UV light.

### Statistical Analysis

GraphPad Prism software was used to analyze the variation between groups. Normal distribution was test using Shapiro–Wilk test. One-way Anova and non-parametric Kruskall–Wallis tests were used to analyze cytokine secretion and parasitic load. Paired *T*-tests were used to analyze flow cytometry stainings. Two-way Anova and Tukey’s multiple comparisons test was used to analyze IgG secretion. For all statistical tests a value of *p* < 0.05 is considered statistically significant and will be followed by the appropriate post-test.

## Results

### *Ex vivo* Tracking of DGNP-DiR

**FIGURE 2:**
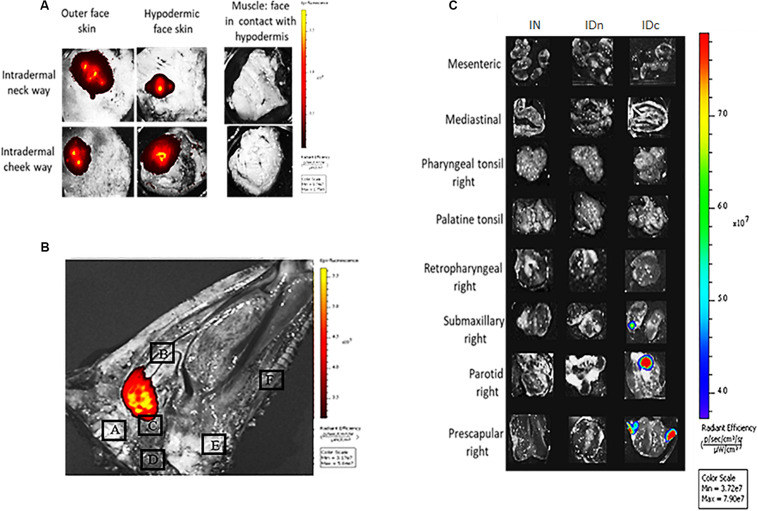
*Ex vivo* tracking of DGNP-DiR administered by intranasal spray or by intradermal inoculation in the neck or in the cheek. DGNP-DiR signal was observed 24 h after administration using the *in vivo* imaging system IVIS^®^ Spectrum (PerkinElmer) under epi-illumination with filters set: 745 nm ex/800 nm em **(A)** Inoculation sites from sheep inoculated with DGNP-DiR by intradermal routes. **(B)** Sagittal cut of head from sheep inoculated with DGNP-DiR by nasal route A: Brain B: Turbinate C: Tubal tonsil D: Medial retropharyngeal lymph node E: Palatine tonsil F: Palate. **(C)** Lymph nodes from sheep inoculated with DGNP-DiR by nasal (IN), intradermal neck (IDn), or intradermal cheek (IDc) route.

To determine the location of the DGNP after administration in the sheep, we performed *ex vivo* tracking of fluorescently-labeled nanoparticles. Two sheep received 600 μg of fluorescently labeled nanoparticles by nasal or intradermal (either in the neck or in the cheek) routes. DGNP-DiR persisted at the sites of inoculation 24 h after administration (Figures 2A,B). Intradermal injected DGNP-DiR were detected on both sides of the skin (Figure 2A). Average radiant efficiency was 5.27e + 08 and 1.22e + 08 in hypodermic face of the neck and the cheek, respectively. No signal in muscle was measured suggesting that intradermal vaccination was effective. As expected for the nasal route (Figure 2B), DGNP-DiR were contained in the pharyngeal septum area close to the pharyngeal tonsils (4.53E + 07). The presence of nanoparticles in relevant draining lymph nodes was then investigated (Figure 2C). 1 h after intradermal neck inoculation, DGNP-DiR were only detected in retropharyngeal lymph node (3.65E + 07; data not shown) and as shown in Figure 2C no more signal was observed after 24 h. While no signal was noticed 1 h after inoculation, 24 h after intradermal cheek inoculation, nanoparticles were detected in submaxillary (5.73E + 07), parotid (9.47E + 07), and prescapular (8.28E + 07) lymph nodes (Figure 2C). At 1 h and at 24 h post-administration, intranasally administered DGNP-DiR were not observed in lymph nodes and were never detected in spleen and in brain whatever the route of administration (data not shown).

### *In vitro* Immunostimulatory Properties of the Vaccine

Induction of a cellular immune response is crucial for the control of *T. gondii*. Notably Th1 cytokines play a critical role for coordinating protective immune response against the protozoa. To determine whether DGNP/TE vaccine induces cellular immune response, Th1 cytokines (IFN-γ and IL-12), Treg cytokine (IL-10), and Th17 cytokines (IL-17) were measured in the supernatant of splenocytes from non-immunized ewes.

**FIGURE 3:**
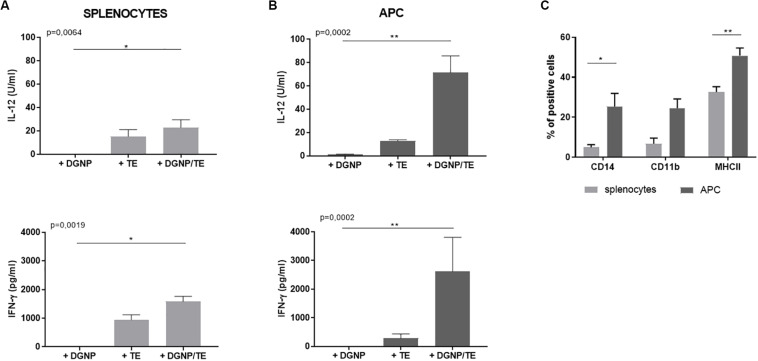
*In vitro* immunostimulatory properties of the DGNP/TE vaccine in non-immunized sheep splenocytes. IL-12 and IFN-γ secreted by **(A)** splenocytes and **(B)** APC enrichment cells from four sheep. Statistical analysis was performed thanks to Kruskall–Wallis test *p < 0.05, **p < 0.01 **(C)** Flow cytometry characterization of splenocytes and APC enriched cells from four sheep. Statistical analysis was performed using paired *t*-test *p < 0.05, **p < 0.01. Data are expressed as mean ± SEM.

As shown in Figure 3A, cells secreted IL-12 and IFN-γ following TE stimulation, 15.11 U/ml (±5.92), and 933.5 pg/ml (±188.2), respectively, while controls did not secrete relevant levels of these cytokines. These cytokine secretions were improved when TE were delivered by DGNP: 22.9 U/ml (±6.57) for IL-12 and 1581 pg/ml (±181.9) for IFN-γ.

In order to analyze the cytokine-secreting cell population involved following the Ags delivery by DGNP, lymphocyte-enriched (non-adherent) and APC-enriched (adherent) fractions from splenocytes were obtained using the adhesive properties of the APC to plastic. Flow cytometry analysis of the non-adherent population did not show any enrichment, whatever the cellular marker (data not shown). Otherwise, adherent population were enriched in CD14 + (37%), CD11b + (25%), and MHC II + (57%) cells compared to splenocytes: 4, 1, and 28%, respectively, (Figure 3C) and impoverished in CD4 + cells (28% to 8% *ns*, data not shown). Therefore, the adherence method probably enriched APC population in CD14+/CD11b + monocytes/macrophages since no significant difference with splenocytes was observed for other markers (CD8, CD11c, or DU204).

When stimulated with DGNP/TE vaccine, APC-enriched cells secreted higher levels of IL-12 (71.37 U/ml ± 14.34) and IFN- γ (2612 pg/ml ± 1195) than total splenocytes. Regarding the cytokine secretion of non-adherent cells, no difference was observed whatever the condition of stimulation (data not shown), supporting the contribution of these APC in the secretion of the cytokines (Figure 3B). No secretion of IL-10 and IL-17 was detected regardless of the conditions (data not shown).

### Humoral Immune Response After Vaccination

In both experiments, *Toxoplasma* specific IgG were explored by endpoint ELISA at day 0, 3 weeks after the first vaccination (D21), 2 months after the second vaccination (D80), and finally 2 months after infection (Figure 1). No specific IgG were detected in the serum of sheep at D0 and D21 whatever the experiment or the route of vaccine administration (data not shown).

**FIGURE 4:**
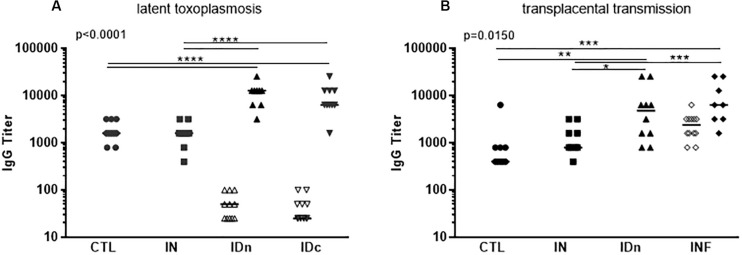
Humoral immune response of sheep following vaccination with DGNP/TE and infection with *T. gondii*. **(A)** Sheep from the latent experiment CTL: inoculated with DGNP alone as control; IN: vaccinated by nasal route; IDn: vaccinated by intradermal route in the neck area; and IDc: vaccinated by intradermal route in the cheek area. **(B)** Sheep from the transplacental transmission experiment CTL: inoculated with DGNP alone as control; IN: vaccinated by nasal route; IDn: vaccinated by intradermal route in the neck area; INF: experimentally primary infected as protected control. Specific anti-*T. gondii* IgG titer were explored by endpoint ELISA at D80 after the first inoculation: **(A, B)** *n* = 12/group (open symbols) and after infection (closed symbols) at the end of experiments **(A)** *n* = 10/group **(B)** CTL *n* = 9; IN *n* = 9; IDn *n* = 10; and INF *n* = 8 infected pregnant sheep. Median and individual data points are shown. Since axis is logarithmic, negative values are not plotted and are <25. Statistical analysis was performed thanks to two-way ANOVA **(A)** p < 0.0001 and **(B)** *p* = 0.0150 and Tukey’s multiple comparisons posttests *p < 0.05, **p < 0.01, ***p < 0.001, and ****p < 0.0001.

As expected, ewes from control group did not develop *Toxoplasma* specific antibodies after inoculation with DGNP alone by either route (Figures 4A,B) in contrast to ewes primary-infected with oocysts (Figure 4B). Also as expected, IgG were detected 2 months after oral infection (400 oocysts of Me49) in control (CTL, Figure 4A) or subcutaneous infection (5.10^6^ tachyzoites of RH) in control and primary-infected groups (CTL; INF, Figure 4B).

Regarding the intradermal routes of vaccination, in the latent toxoplasmosis experiment, sheep developed a mild, specific *Toxoplasma* humoral response, with median titer IDn = 50 and IDc = 25 compare to CTL and IN < 25 (Figure 4A). In contrast, in the transplacental experiment, IgG were no longer detected in sheep vaccinated twice by the intradermal neck route (IDn, Figure 4B). 2 months after challenges, all sheep from ID group developed specific IgG without difference with primary-infected (INF) sheep (Figures 4A,B).

Concerning the nasal route of vaccination (IN), IgG were never detected after one or two administrations of DGNP/TE. 2 months after challenge, a specific IgG secretion was observed (IN, Figures 4A,B) without difference with CTL, but lower compared with the intradermal neck route and challenged (IDn, Figures 4A,B) or in primary-infected and challenged (INF, Figure 4B).

To conclude, intranasal vaccination of sheep with DGNP/TE vaccine did not induce serum-IgG production. Experimental challenge led to specific IgG production that was significantly lower than observed in sheep vaccinated by intradermal routes or primary-infected.

### Cellular Immune Response After Vaccination

To determine whether vaccination *via* transdermal and/or nasal routes with the DGNP/TE vaccine induced a cellular immune response, *Toxoplasma* specific cellular proliferation and cytokine secretions were analyzed in the supernatant of TE-restimulated cells from spleen, lymph nodes and PBMC (Figure 1). For the phenotyping of spleen and lymph nodes cells there is no TE-stimulation *in vitro*.

Peripheral Blood Mononuclear Cells *compartment*: For both experiments, PBMC of all ewes were isolated at day 0 and day 28 (7 days after the second vaccination). No cellular proliferation in response to specific *Toxoplasma* antigens stimulation was observed. Moreover, despite specific IFN-γ and IL-17 secretion by PBMC from sheep vaccinated by intradermal neck and intranasal routes, heterogeneity of the IFN-γ and IL-17 responses in all groups, especially at day 0 and for the control group, made interpretation impossible (data not shown).

#### Lymph Node Compartment

**FIGURE 5:**
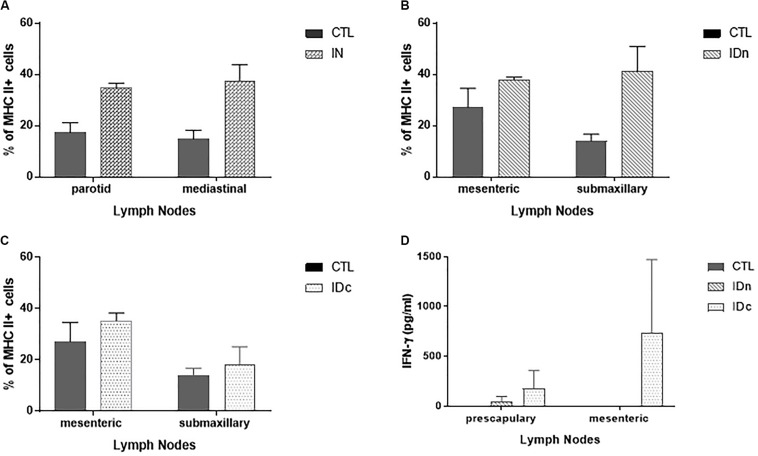
Cellular immune response in draining lymph nodes of sheep (2 per group) 6 months after the last vaccination in the latent toxoplasmosis experiment. Data are expressed as mean ± SEM. **(A)** Expression of MHCII + cells among draining lymph nodes cells from intranasally immunized ewes (IN) compared to CTL. **(B)** Expression of MHCII + cells among draining lymph nodes cells from ewes vaccinated by intradermal route in the neck area (IDn) compared to CTL. **(C)** Expression of MHCII + cells among draining lymph nodes cells from ewes vaccinated by intradermal route in the cheek area (IDc) compared to CTL. **(D)** Specific IFN-γ secretion in draining lymph nodes from ewes vaccinated by both intradermal routes compared to CTL.

Lymph nodes (submaxillary, parotid, retropharyngeal, prescapular, mediastinal, mesenteric, pharyngeal, and palatine tonsils) from two ewes per group of latent toxoplasmosis experiment were collected 6 months after the final immunization. As shown in Figure 5, over-expression of MHCII was seen in the draining lymph nodes of the corresponding administration site compared to control group (Figures 5A–C). This over-expression was not observed for CD4, CD8, CD11b, and CD11c staining. While the vaccine did not modulate cell proliferation after 48 h of stimulation with 20 μg/ml of TE (data not shown), specific IFN-γ production was noticed in prescapular and mesenteric lymph nodes from ewes immunized by both intradermal routes (Figure 5D); this was not observed for any draining lymph nodes of the IN group, nor in the other lymph nodes of the ID groups.

#### Spleen Compartment

**FIGURE 6:**
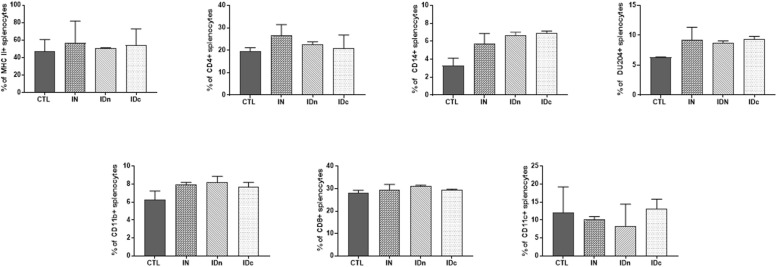
Expression of immune markers of splenocytes from sheep (2 per group) 6 months after the last vaccination in the latent toxoplasmosis experiment. Splenocytes were isolated and stained for the immune markers MHCII, CD4, CD14, DU204, CD11b, CD8, and CD11c. CTL: inoculated with DGNP alone as control; IN: vaccinated by nasal route; IDn: vaccinated by intradermal route in the neck area; and IDc: vaccinated by intradermal route in the cheek area. Data are expressed as mean ± SEM.

Spleens from two ewes per group of the latent toxoplasmosis experiment were collected 6 months after the final immunization. Regarding cells recruitment in spleen, an increase of MHCII (+10%) and CD4 (+7%) expression was observed for intranasally vaccinated sheep-splenocytes only, compared to control (Figure 6). No difference was observed between groups for DU204, CD14, CD11c, CD11b, and CD8 staining.

**FIGURE 7:**
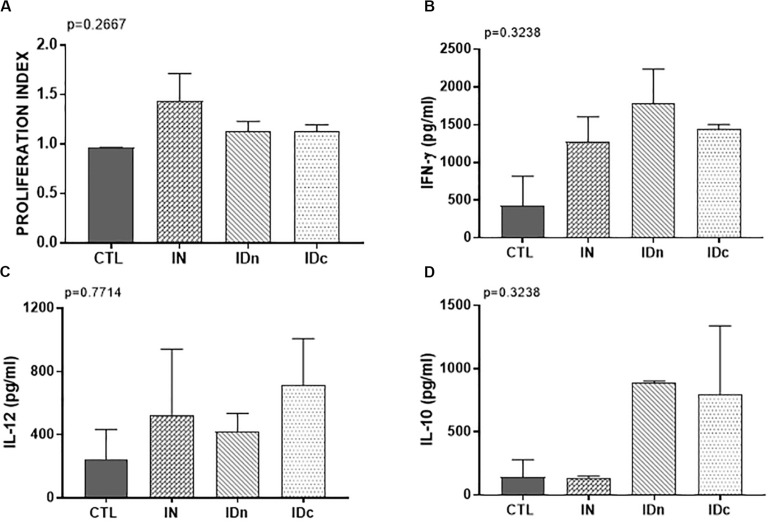
Cellular immune response from spleen of two sheep 6 months after the last vaccination in the latent toxoplasmosis experiment. CTL: inoculated with DGNP alone as control; IN: vaccinated by nasal route; IDn: vaccinated by intradermal route in the neck area; and IDc: vaccinated by intradermal route in the cheek area. **(A)** Proliferation index of spleen cells measured by colorimetric BrdU. **(B)** IFN-γ secretion by spleen cells. **(C)** IL-12 secretion by spleen cells. **(D)** IL-10 secretion by spleen cells. Data are expressed as mean ± SEM. Statistical analysis was performed using Kruskall–Wallis test and was non-significant.

A *Toxoplasma* specific proliferation was only observed for splenocytes from sheep vaccinated by nasal route (IN; Figure 7A). After TE stimulation, spleen cells from all immunized ewes (IN, IDn, and IDc) secreted more IFN-γ (1500 pg/ml, Figure 7B) and IL-12 (550 pg/ml, Figure 7C) than control (430 pg/ml and 245 pg/ml, respectively). IL-10 production was not increased in the nasal route group (140 pg/ml) but was largely greater in both groups vaccinated by intradermal routes (840 pg/ml, Figure 7D). No secretion of IL-17 was detected regardless of conditions (data not shown). These results showed that immunization, whatever the route, tend to induce Th1-related cytokines associated with protection. The only difference between the nasal and the intradermal route is the IL-10 secretion suggesting that the response may be more regulated by this route and may be linked to the lack of protection.

### Detection of *Toxoplasma* in Brain

In order to evaluate the protection conferred by the DGNP/TE vaccine, challenge was performed 2 months before brain cyst counting in sheep or lambs in both experiments (Figure 1).

### Latent Toxoplasmosis Experiment

**FIGURE 8:**
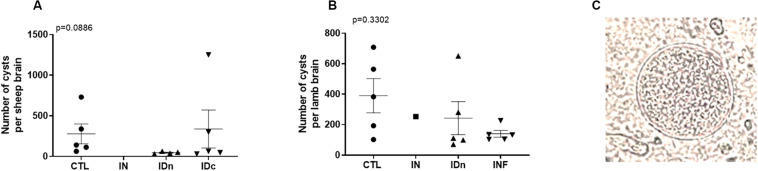
Brain cysts numeration from **(A)** latent toxoplasmosis experiment and **(B)** transplacental transmission experiment. Brain cysts load were calculated related to their individual total volume. Data are expressed as plot and mean ± SEM. Statistical analysis was performed using Kruskall–Wallis test and was non-significant. **(C)** Representative picture of cyst observed with an optical microscope at an original magnification of x400.

Two months after challenge, no cysts were detected in the brains of sheep vaccinated by nasal route (IN: 0/11), contrary to control sheep (CTL: 5/8 positive brain), or to sheep vaccinated by both intradermal routes (IDn: 4/9 and IDc: 5/11 positive brain; Table 1). For these cyst-negative brains, the parasitic load was below the detection limit. For the cyst-positive brains (Figure 8A), no significant difference was observed between groups due to the heterogeneity of results (overall *p* = 0.0886; IDc: min 31 to max 1250). However, a tendency to decrease by about 62% was observed in groups vaccinated by intradermal routes relative the control group (CTL: 141 *vs* IDn: 44 and IDc: 63). Therefore, only sheep vaccinated twice by the intranasal route were fully protected against the oral challenge with *Toxoplasma* oocysts.

**TABLE 1:** Number of cyst-positive brains from latent toxoplasmosis experiment (on the left) and transplacental transmission experiment (on the right).

	CTL	IN	IDn	IDc		CTL	IN	IDn	INF
Negative brains	3	11	5	6	Negative brains	4	7	3	3
Positive brains	5	0	4	5	Positive brains	5	1	5	5
% positive brains	62.5%	0%	44.4%	45.4%	% positive brains	55.5%	12.5%	62.5%	62.5%

*Number of brain where cysts were detected (positive brain, see Figure 8) or not (negative brain < detection limit).*

#### Transplacental Transmission Experiment

Counting of lamb brain cysts occurred 1-month after delivery. As shown in Table 1, the number of cyst-positive lamb brains from non-vaccinated group (CTL), intradermal vaccinated group (IDn), or primary infected group (INF) were approximately the same: 5/9 (55%), 5/8, and 5/8 (62%), respectively. Concerning lambs from intranasally vaccinated group (IN), only one brain (12%) was positive among eight. For cyst-positive brains (Figure 8B), no significant differences were observed between groups due to heterogeneity of results (overall *p* = 0.3302; IDn: min 70 to max 651) but a tendency to decrease the number of cyst per brain was still observed in two vaccinated groups (IN: 253, IDn: 111) or the primary-infected animals (INF: 132) *vs* control (CTL: 383).

Although Toxoplasma DNA has been detected in almost all brains, cysts were not detected by direct counting in the brains of lambs from nasal immunized ewes (contrary to primary-infected group). Results were consistent with a very strong reduction of parasitic load after nasal immunization. Moreover, it should be note that neither of the two techniques has demonstrated the presence of the parasite in the brain of one lamb from ewes vaccinated by intranasal route.

## Discussion

Toxoplasmosis is a zoonotic infection with global impact. Considered as one of the most important illnesses associated with foodborne hospitalizations and deaths in humans, toxoplasmosis is also a major cause of reproductive losses in small ruminants, both farmed and otherwise, worldwide.

Currently, there is only one commercial vaccine (Toxovax^®^) licensed for use to protect against congenital toxoplasmosis in ewes (20). If Toxovax^®^ – based on the live-attenuated S48 strain – is highly effective, it presents several significant drawbacks: it is expensive, is not without adverse effects, has a short shelf-life, and persists as tissue cysts in the brains of the vaccinated sheep which could potentially revert to a pathogenic strain. This vaccine is not suitable for human use, therefore, and there is currently no licensed human vaccine available (22). As vaccination against toxoplasmosis appears to be the most cost-effective preventive measure, in terms of both veterinary and human health, a One Health vaccination approach could be promising in order to reduce the impact of *T. gondii*.

In recent years, we have developed an innovative, mucosal antigen-delivery platform to produce an anti-*Toxoplasma* vaccine for both animal and human applications, according to the One Health concept. Our approach has centered on a stable, efficient and adjuvant-free vaccination approach based on the use of biobased nanoparticles for mucosal delivery. These nanoparticles are made of starch and lipids and are able to carry all the antigens of *Toxoplasma* (DGNP/TE) for cellular delivery. The proof-of-concept of this vaccine has already been performed in both chronic and congenital toxoplasmosis in a murine model. Animals which received the vaccine *via* nasal route developed a specific and robust Th1 and Th17 cellular immune response at spleen level and were fully protected against oral challenge mimicking natural infection. In parallel nanoparticles were totally cleared into the gastrointestinal tract within hours and then eliminated in the feces, reflecting the safety of this vaccine approach (38, 39).

Here, we wanted to validate the efficiency of the vaccine in sheep since it is a highly prevalent disease in this ruminant and, beyond the health concerns, it has a significant economic impact. Indeed, toxoplasmosis is an important cause of reproductive losses in sheep (as well as more widely for the livestock industry). Moreover, sheep are also a relevant animal model for human vaccine development: 1- especially for the development of nasal vaccine since the structure of their nasal cavities is similar (42); 2- because congenital diseases in the two species display strong similarities. After primary infection, both ewes and women develop protective immune response preventing transplacental transmission following re-infection (43). This common anti-*Toxoplasma* immunity involved a strong IL-12 production by APCs, inducing Th1-polarized CD4 and robust CD8 T cell effectors as important source of IFN-γ (28, 44), the major mediator of resistance against *T. gondii.*

There are several drawbacks in using live-attenuated *Toxoplasma* vaccine since it could generate infectious cysts in meat destined for consumption, especially lamb, or in humans. Therefore, inactivated vaccines are to be preferred, but they are generally less immunogenic and have been shown to be not completely protective in sheep (45, 46). These issues have led to the emergence of subunit vaccines associated with particulate delivery systems, in particular immune-stimulating complexes (ISCOMS) encapsulating *T. gondii* membrane antigens, and poly(lactide-*co*-glycolide) micro- and nanoparticles formulated with crude extract of *T. gondii* tachyzoites adjuvanted with cholera toxin. However, these approaches reported no protection in sheep following oral challenge with *T. gondii* (23, 24).

In this study, we investigated the capacity of the DGNP/TE vaccine to induce a protective immune response against chronic and congenital ovine toxoplasmosis and we evaluated two different immunization routes (intranasal and intradermal) on the induction of protective immune responses at the systemic and mucosal levels.

We analyzed the persistence of nanoparticles for at least 24 h at inoculation sites and in draining lymph nodes. As demonstrated in mice (35), we here confirm that intranasally-administered nanoparticles do not migrate through the mucosa to the draining lymph nodes and are not observed in the brain. Again, we have provided evidence of the ability of these nanoparticles to prolong antigen release to immunocompetent cells. *In vitro* we studied the immune-stimulatory efficiency of DGNP/TE vaccine on ovine splenocytes. We observed that DGNP alone and free TE did not stimulate cells as no cytokines (IL-12, IFN-γ, and IL-10) were observed. In contrast, a significant increase of IL-12 and IFN-γ was obtained for splenocytes activated by the DGNP/TE formulation, with no secretion of IL-10, together suggesting the DGNP/TE vaccine was able to stimulate pro-inflammatory cytokines, important for acquiring *Toxoplasma* immunity. To identify more precisely the cellular source of this secretion, we collected APC-enriched fractions (CD14+, CD11b+, and MHCII+) and observed even greater secretion of IL-12 and IFN-γ. It is likely that these CD14+ and CD11b + cells correspond to the sub-population recently identified by Ferret-Bernard et al. (47), described as presenting a prominent capacity to produce these cytokines in response to stimulation.

*In vivo*, specific-antibodies lack against *T. gondii* was exhibit for intra-nasally vaccinated sheep contrary to few responses for intradermic administration. Stanley et al. (24) in a similar experiment using PLGA NP via intranasal route loaded with total parasite extract detected very little antigen-specific seric IgG antibody and no IgG in nasal secretions without real explanation. In a recent publication, intramuscular and intranasal forms of administration of anti-Fasciola vaccine were applied in sheep. Intramuscular vaccination was able to induce a strong systemic antibody response against fluke antigens but failed to confer significant protection. No significant systemic or localized specific antibody response was detected following nasal vaccination, although a strong and rapid antigen-specific IgG response was observed following challenge, indicating vaccination had primed the host immune system (48).

However, we showed that ewes vaccinated *via* the intranasal route were better protected against an experimental toxoplasmosis challenge compared with intradermal-immunized sheep, emphasizing the relevance of intranasal route of vaccination in providing protective immunity against *Toxoplasma* infection. Despite IFN-γ and IL-17 secretion by PBMC, it was not possible to conclude due to heterogeneity between animals. IFN-γ heterogeneity was also observed by Verhelst (30). In this work, IFN-γ was only detected at 8–10 dpi in PBMC but only in half tested animals. However, cellular and molecular signatures in spleen and secondary lymph nodes were quite similar between the two routes of vaccination and cannot predict the vaccine efficacy; signs of vaccination-triggered immune activation were observed in the spleen *via* secretion of IL-12 and IFN-γ by APC cells. The only difference between the two routes of administration was the IL-10 secretion by splenocytes of ID immunized sheep (not seen in IN sheep), suggesting that IL-10 could participate in favoring infection. Indeed, several studies have demonstrated that IL-10 production secreted during toxoplasmosis decreased host resistance and increased parasite burden (49). Concerning cells distribution following vaccination, increase of MHCII + cells in spleen and lymph nodes, especially for sheep vaccinated by intranasal route, may reflect induction of a cellular response suggesting that vaccination is effective. Surprisingly, no mucosal cell-mediated immunity was detected following intranasal administration and we found no correlation with protection. Comparable results were obtained in our mouse model in the absence of a mucosal immune response induced by intranasal vaccination (38).

In conclusion, this study suggests not only that DGNP/TE are a relevant vaccine delivery platform to immunize against latent and congenital ovine toxoplasmosis, but also that the intranasal route of vaccination proffers better protection than the intradermal route. Our data support the intranasal route of vaccination for a toxoplasmosis vaccine under development for potential human applications.

## Data Availability Statement

All datasets presented in this study are included in the article/Supplementary Material.

## Ethics Statement

The animal study was reviewed and approved by Comité d’Ethique en Expérimentation Animale Val de Loire Dependent on the Ministry of Higher Education, Research and Innovation. Written informed consent was obtained from the owners for the participation of their animals in this study.

## Author Contributions

DB, ID-P, and CD designed the study. CH performed vaccine formulation. CD and NM performed the experiments, acquired, and analyzed the data. IL performed, acquired, and analyzed all imaging data. PC realized the humoral response analysis for the revision of the manuscript. CD and ID-P wrote the manuscript. NM, RC, and DB revised the original draft. All authors contributed to the article and approved the submitted version.

## Conflict of Interest

DB is founder, CEO, and CSO of VAXINANO. CH is employee of VAXINANO. The authors declare that this study received funding from Vaxinano, SAS. The funder had the following involvement in the study: collaborative design of the project and revision of original draft.
