# Supplementary figures and images for: Effective Nanoparticle-Based Nasal Vaccine Against Latent and Congenital Toxoplasmosis in Sheep
Source: Front Immunol. 2020 Sep 9;11:2183. doi: 10.3389/fimmu.2020.02183 (PMC7509486; doi:10.3389/fimmu.2020.02183)

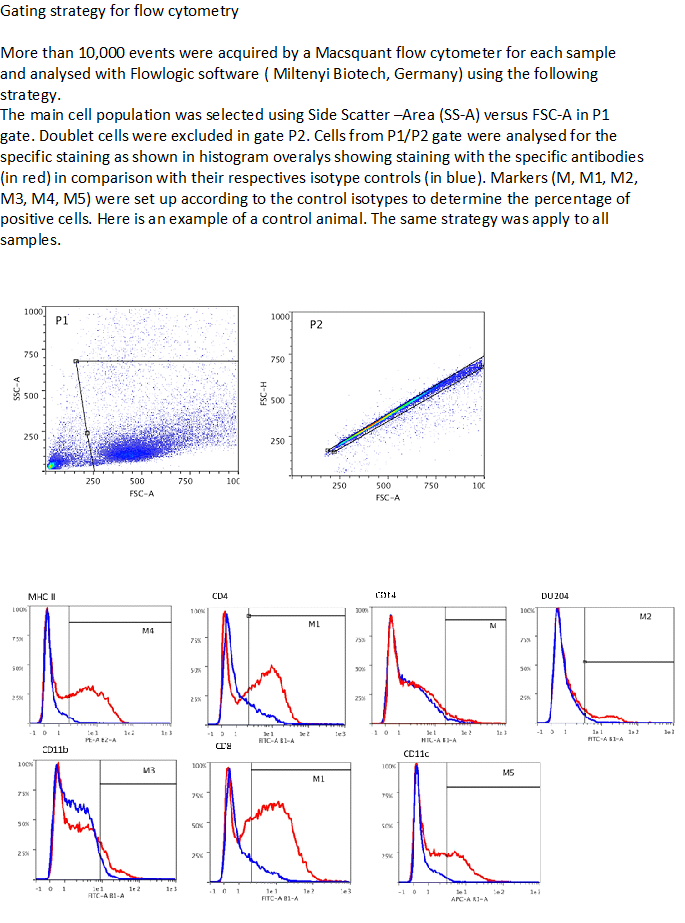

Supplement: Supplementary file 1 [file Image_1.TIF]
